# Perforator plus flaps: Optimizing results while preserving function and esthesis

**DOI:** 10.4103/0970-0358.73425

**Published:** 2010

**Authors:** Sandeep Mehrotra

**Affiliations:** Sr Adv (Surgery& Plastic), Command Hospital (Central Command), Lucknow 226 002

**Keywords:** Function and form preservation, perforator plus flaps

## Abstract

**Background:**

The tenuous blood supply of traditional flaps for wound cover combined with collateral damage by sacrifice of functional muscle, truncal vessels, or nerves has been the bane of reconstructive procedures. The concept of perforator plus flaps employs dual vascular supply to flaps. By safeguarding perforators along with supply from its base, robust flaps can be raised in diverse situations. This is achieved while limiting collateral damage and preserving nerves, vessels, and functioning muscle with better function and aesthesis.

**Materials and Methods:**

The perforator plus concept was applied in seven different clinical situations. Functional muscle and fasciocutaneous flaps were employed in five and adipofascial flaps in two cases, primarily involving lower extremity defects and back. Adipofascial perforator plus flaps were employed to provide cover for tibial fracture in one patients and chronic venous ulcer in another.

**Results:**

All flaps survived without any loss and provided long-term stable cover, both over soft tissue and bone. Functional preservation was achieved in all cases where muscle flaps were employed with no clinical evidence of loss of power. There was no sensory loss or significant oedema in or distal to the flap in both cases where neurovascular continuity was preserved during flap elevation. Fracture union and consolidation were satisfactory. One patient had minimal graft loss over fascia which required application of stored grafts with subsequent take. No patient required re-operation.

**Conclusions:**

Perforator plus concept is holistic and applicable to most flap types in varied situations. It permits the exercise of many locoregional flap options while limiting collateral functional damage. Aesthetic considerations are also addressed while raising adipofascial flaps because of no appreciable donor defects. With quick operating times and low failure risk, these flaps can be a better substitute to traditional flaps and at times even free tissue transfers.

## INTRODUCTION

The cornerstone of reconstructive work is the provision of wound flap cover which requires a delicate balance between vascularity and reach, while limiting collateral damage. It is desirable to provide wound cover without sacrifice of functional muscles, neurovascular supply, or aesthetics.[1,2]

Traditional flaps, at times, suffer the disadvantage of flap ischemia and loss over critical areas. Free flaps offset this problem but are technically demanding, time consuming, and equipment intensive. Perforator anatomy and mapping allow diverse locoregional fasciocutaneous flaps albeit with limitations imposed by perforator location and size. The concept of perforator “plus” flaps aims to offset the disadvantages of traditional and perforator flaps while increasing applicability. Traditional fasciocutaneous flaps have vascular supply only from the base whereas true perforator flaps are island flaps based on a single perforator. By marrying the two concepts, perforator “plus” flaps are those which are designed based on a perforator input “plus” additional vascular supply from a retained base.[3] These are robust, have high success rates and can be employed not only in emergency settings, but also in the presence of infection and hitherto contraindicated situations. Preservation of sensory nerves, subcutaneous arteries, and veins during flap elevation is often considered inconsequential. The resulting persistent lower limb/flap edema and nagging sensory loss with insensate flaps are, however, preventable. The dual blood supply of perforator plus flaps with a wide safety margin, permits variations, and prevents sacrifice of functioning muscle or neurovascular supply.

The perforator plus concept was applied to various flap types including adipofascial, muscle, and myocutaneous. In all cases, primary defect closure was obtained without flap failure in any patient. Morbidity and collateral damage were minimized without compromising on end result, wound stability, or fracture union.

## MATERIALS AND METHODS

The defects were assessed clinically and possible traditional flaps in the vicinity were considered based on the availability of regional muscle, adipofascial, or skin. Flaps were chosen based on the principle of minimal morbidity and collateral damage. It was attempted to avoid functioning muscle sacrifice, preserve cutaneous nerves/vessels, and follow esthetic needs. Known perforators within the flap circumference were located by a handheld Doppler. Limb surgeries were performed under tourniquet control without exsanguination. Perforator dissection was performed by the naked eye/loupe using microsurgical instruments from the defect margins till the selected perforators were identified, mobilized, and flap raised. The flap base was always retained and narrowed if required to permit tensionless inset. Detached muscles were reinset after mobilization and achieving wound cover to minimize/avoid functional deficit. Creation of secondary defects was avoided, and immediate grafting of residual defects was done. Subcutaneous vessels and nerves underlying the flap were preserved in continuity while retaining inputs to the flap itself. Suction drains were employed on need basis. No special flap monitoring techniques or drugs were employed in any case.

## RESULTS

Seven patients underwent perforator plus flaps based on posterior trunk, inguinal region, and lower extremity trauma. The age ranged from 28 to 67 years with a mean of 44 years. Follow-up was on an average of 11 months (range, 1–13 months). Three flaps were myocutaneous perforator plus flaps where the muscle origin/insertion were retained as the additional blood supply along with perforator inputs. One sliding muscle flap employed tibialis anterior (TA) and flexor digitorum longus (FDL), retaining segmental perforators while shifting muscle origins. Two lower extremity perforator fasciocutaneous flaps were raised without sacrifice of saphenous neurovascular bundles passing through their substance. There was no sensory loss distal to the flap and no clinically apparent distal limb oedema. Retained neurovascular inputs to the flap limited their oedema and resulted in sensate flaps. The remaining two cases underwent perforator plus adipofascial flaps with subdermal dissection and no consequent secondary defects. The adipofascial flap peninsular base was retained while the preserved perforator provided the main vascular input. None of the flaps exhibited loss and stable wound cover was achieved in all on follow-up. One adipofascial flap exhibited minor graft loss due to β-hemolytic streptococcus wound infection. Stored graft was applied with subsequent successful graft take. Operative time averaged 130 min (range, 90–180 min). Exposed fracture sites exhibited sound bony union with no evidence of osteomyelitis and none required re-operation. One patient expired 1 month postoperatively due to uncontrolled iliofemoral bleed from metastatic penile carcinoma.

## CASE REPORTS

### Case 1

**Figure 1:**
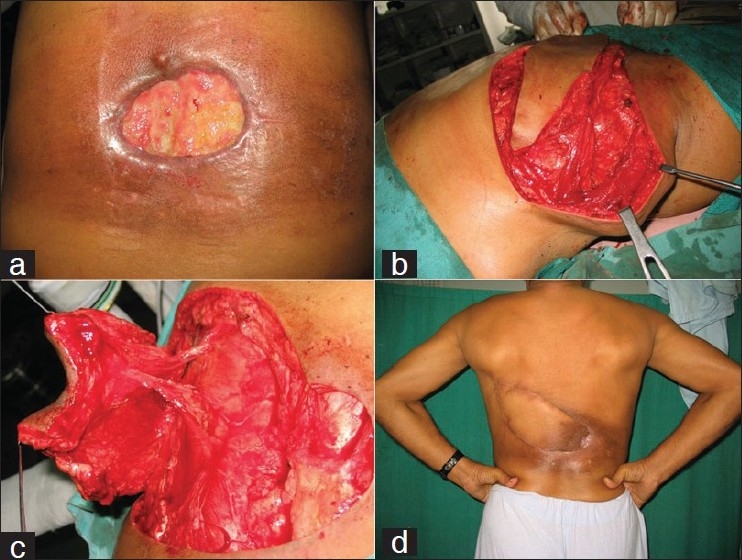
Case 1: A 48-year old man with post-tumor excision defect over back. (a) The postradiotherapy changes are evident. (b) Skin paddle raised over superior segment of right Latissmus dorsi. (c) Lateral paraspinal perforators preserved by intramuscular dissection. (d) V-Y flap advancement and primary closure with stable long-term cover and preserved muscle function.

A 48-year veteran presented with a 15 × 7 cm^2^ defect overlying thoracolumbar spine of 6 months duration resulting postexcision and radiotherapy for soft tissue sarcoma. Two attempts elsewhere for wound closure and grafting failed due to the ischemic ulcer. A muscle “fill” and overlying stable sensate skin cover was deemed essential. Lateral paraspinal perforators were marked by Doppler, the medial row of lumbar perforators being sacrificed during tumor extirpation. The ulcer was excised and a V-Y skin flap was raised over the superior segment of *Latissmus dorsi*. The paravertebral muscle origin was detached and intercostal perforators and nerves dissected intramuscularly. Segmental dissection preserved thoracodorsal vessels and nerve and permitted movement of the entire superior segment of latissmus with the overlying skin paddle into the defect. The muscle origin was resutured across the midline and the skin closed in a V-Y advancement while leaving the humeral insertion intact. The blood supply to the Latissmus was retained both from the thoracodorsal trunk as well as lateral paraspinal perforators. Preservation of intercostals nerves resulted in sensate cover. Healing was uneventful, and the patient noted no discernable deficit of shoulder power or movement. On follow-up at 12 months, the cover remained stable [Figure 1]

### Case 2

**Figure 2:**
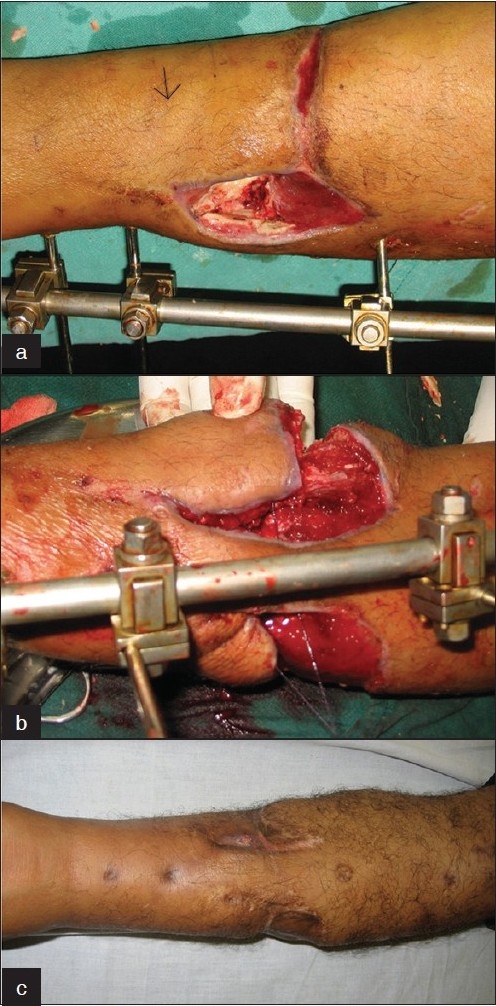
Case 2: (a) A 28-year old man with comminuted fracture tibia middle third. The arrow indicates the posterior tibial perforator. (b) A distally based skin flap is elevated along with mobilized FDL to protect the perforator while covering the exposed fracture. (c) One-year postoperative with consolidated fracture.

A 28-year soldier sustained a compound comminuted fracture lower third left leg in a road accident and was referred 7 weeks later with exposed bones at the fracture site. Two posterior tibial perforators were identified and a distally based perforator plus flap planned due to a deep proximal laceration [Figure 2]. The skin was densely adherent to the underlying muscle precluding subfascial dissection. The underlying FDL muscle was effaced from the tibial origin safeguarding its perforators and with the overlying skin, slid into the bony defect. Healing was uneventful, and the external fixator was replaced by IM nailing. Follow-up at 15 months revealed sound bony union with stable cover and active toe flexion.

### Case 3

**Figure 3:**
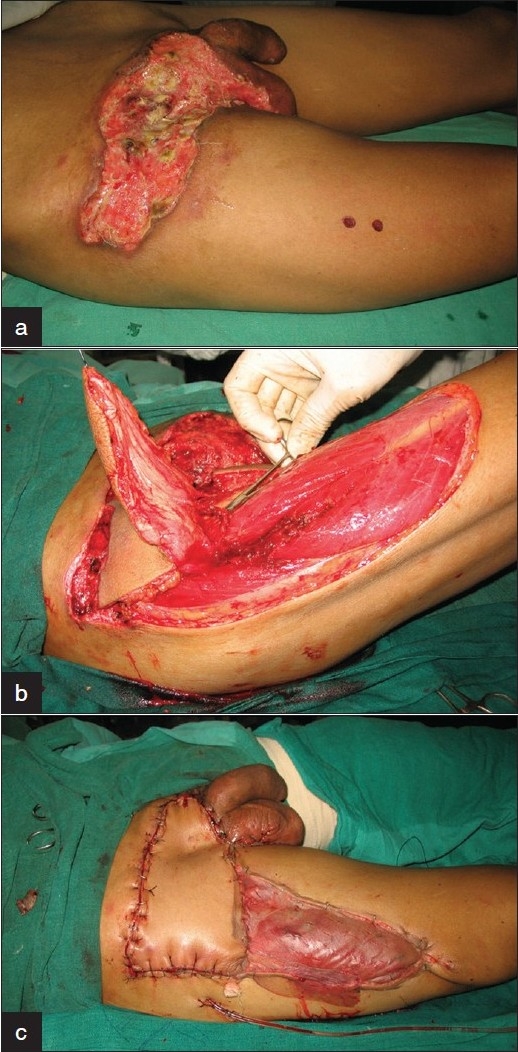
Case 3: (a) Extensive metastatic disease right groin from penile carcinoma in a 36-year old man with exposed femoral vessels. The anterolatral thigh perforators are marked. (b) Musculocutaneous flap raised preserving dual supply from origin of tensor fascia lata and upper thigh perforator. The lower perforator has been ligated. (c) Flap inset and split skin grafting of secondary defect.

A 36-year soldier was referred with a progressively enlarging malignant ulcer in the right groin of 25 × 9 cm^2^ extending from penile base till right ilium. The femoral vessels were exposed and covered with slough with risk of impending rupture. Histopathological examination confirmed metastatic penile carcinoma. Doppler revealed two anterolateral thigh perforators in close proximity. After careful debridement of the ulcer, an anterolateral thigh flap was raised including the tensor fascia lata origin. The proximal perforator was dissected along its pedicle after ligation of the distal one. Adequate perforator length permitted flap rotation by 110° based on vascular supply both from the perforator and muscle origin. The secondary defect was split skin grafted [Figure 3]. The patient was reffered to an oncology center where he underwent salvage therapy for his malignancy, but expired 4 weeks later due to inexorable tumor growth and massive hemorrhage from iliofemoral vessels.

### Case 4

**Figure 4:**
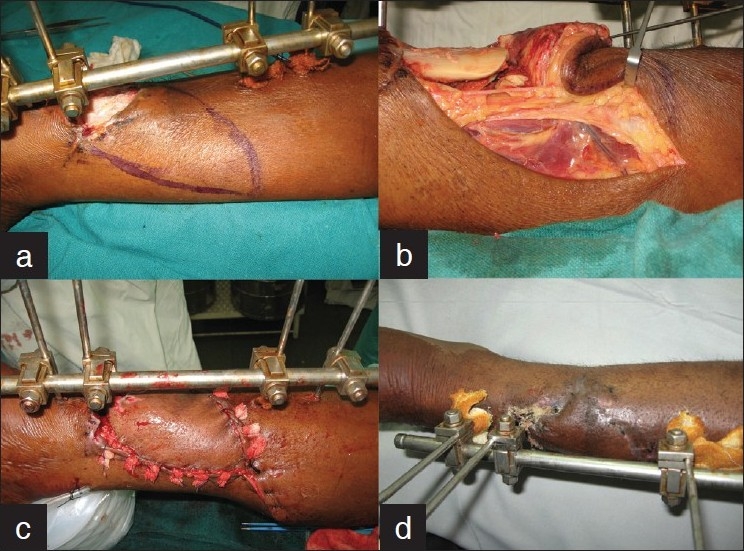
Case 4: (a) Exposed comminuted lower third tibial fracture. (b) A posterior tibial perforator based V-Y advancement flap raised. The preserved saphenous neurovascular bundle is passing through the flap distally to the foot. (c) V-Y advancement done retaining flap neural and perforator input. (d) Three weeks postoperative with negligible edema of flap and distal limb.

A 47-year old diabetic suffered multiple fractures right leg due to collapse of a wall under construction. The compound comminuted lower third tibial fracture was stabilized with an external fixator and wound debrided. He was referred 4 weeks after the injury with an exposed fracture site. A fasciocutaneous medial leg V-Y perforator advancement flap was planned based on posterior tibial artery perforator. The flap was elevated on the perforator while preserving the underlying saphenous neurovascular bundle [Figure 4]. Dissection proximal and distal to the flap around the neurovascular bundle resulted in significant reach to advance and the inset the flap without tension or secondary defect. The flap healed without complications. Four months follow-ups show evidence of stable cover with bony union in progress.

### Case 5

**Figure 5:**
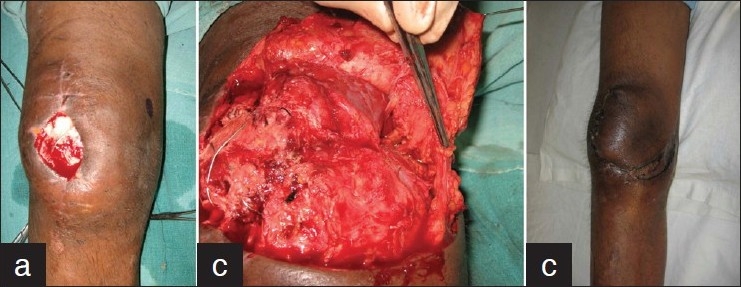
Case 5: (a) Fracture patella with exposed hardware and identifi ed medial perforator. (b) A fasciocutaneous “perforator plus” flap has been elevated safeguarding the perforator emerging under the vastus lateralis and saphenous neurovascular bundle passing medially through it. (c) No distal limb or flap edema 3 weeks postoperatively.

A 48-year security personnel sustained a fracture of the right patella following a fall. Tension band fixation was done following which he had wound breakdown. He was referred 5 weeks later with a 2″ × 2″ open wound over the knee with exposed patella and hardware. A medial knee perforator was marked and proximally based fasciocutaneous flap elevated. The underlying perforator was safeguarded while preserving and maintaining the continuity of the great saphenous vein, saphenous artery, and nerve [Figure 5]. Neurovascular mobilization proximal and distal to the flap permitted its transposition into the defect without tension on the saphenous bundle or the flap. The wound healed rapidly with no evident flap or distal limb edema. The patient did not manifest any sensory loss over the flap or distal limb and has stable sensate cover over the knee on follow-up at 13 months.

### Case 6

**Figure 6:**
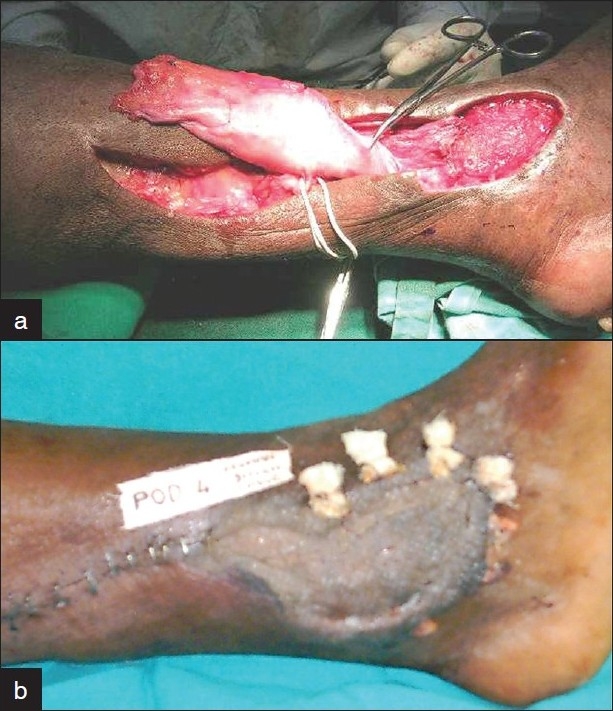
Case 6: (a) A 67-year old diabetic with chronic venous ulcer left ankle. Lower posterior tibial perforator plus adipofascial turnover flap. (b) Postoperative result with consolidating graft and no secondary defect.

A 67-year diabetic presented with a nonhealing venous ulcer over left medial ankle of 8 months duration. He had refused surgery for varicose veins and was on conservative therapy for the same with no deep vein thrombosis. Conservative therapy for the ulcer and split skin grafting elsewhere had been unsuccessful. He had a 5 × 3 cm^2^ indolent ulcer with pale granulation. The lower medial tibial perforator was localized just above the ulcer. A turnover fascial flap was dissected subdermally from the medial leg skin and based distally. The perforator and distal flap base of the adipofascis were retained. This dual supply flap was inset into the defect and grafted. The elevated skin flaps were primarily closed leaving no secondary defect [Figure 6]. Graft take was complete with satisfactory healing and no recurrence at 4 months follow-up.

### Case 7

**Figure 7:**
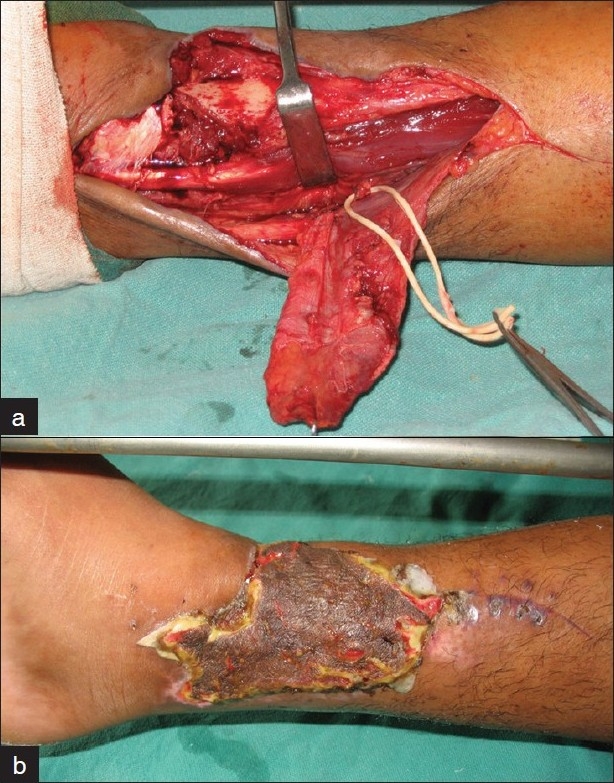
Case 7: (a) A 37-year old man 3 weeks following compound comminuted fracture lower end tibia. A lower posterior tibial perforator plus adipofascial flap has been elevated to cover the exposed bone. (b) Four weeks postoperative with stable cover and no secondary defect.

A 37-year soldier fell from a truck and sustained a comminuted fracture of the lower end right tibia. After debridement and skeletal fixation, he was referred 3 weeks after the injury with a 6 × 8 cm^2^ raw area in the lower leg with exposure of 5 cm tibia devoid of periosteum. Wound culture was positive for β-hemolytic streptococcus. Two perforators were identified by Doppler along the medial leg. The patient was operated 5 weeks after the injury after a negative culture report. An adipofascial perforator flap was planned and subfascial dissection done along the medial wound edge till the perforators were identified and mobilized. Dissection was then completed till the earmarked flap margins. Subcutaneous dissection separated the medial skin from the underlying fascia. Scissors inserted through stab incisions at flap margins permitted adipofascial flap mobilization without devascularization of the skin. A superiorly based adipofascial transposition flap was raised and inset after ligating the lower perforator [Figure 7]. The fascia was split skin grafted and skin incisions closed. Postoperatively there was evidence of cellulitis with wound infection and graft loss. Culture revealed infection with β-hemolytic streptococcus. The adipofascial flap, however, survived in entirety. Antibiotics were continued till the swab report was negative prior to application of stored graft. The subsequent graft take was complete and a stable cover was achieved.

## DISCUSSION

Attention to regional flap applied anatomy has been refocused with the advent of perforator flaps.[4] These flaps are promising in terms of donor site morbidity and accuracy, being likely first choice in the near future.[5] The evolving concept of “perforator plus” flaps has added to the role of locoregional flaps. From difficult situations in lower limb trauma to the management of pressure sores, these flaps are finding increasing use in diverse situations with reproducible good results.[6,7]

The *Latissmus dorsi* is a versatile reliable flap with varied uses. Large spinal defects closure has been variously described by Desprez *et al*.,[8] Moore *et al*.,[9] and McCraw *et al*.[10] Variations of bipedicle advancement were employed including V-Y closure or lateral skin grafting with 5% dehiscence rate. Other latissmus flaps have skin islands on standard or reversed muscle leading to its function sacrifice. The latissmus flap in case 1 was employed while preserving vascularity, nerve supply, and function. Paraspinal perforators were dissected and retained to allow 12 cm shift of muscle origin across the midline. The islanded skin was advanced in a V–Y fashion to provide stable cover after the muscle filled in the radiation defect and no secondary defect resulted.

Essential to lower extremity salvage trauma protocols is adequate and early soft tissue coverage requiring use of vascularized locoregional or free flaps.[11] Exposure of bone, hardware or tendons poses specific reconstructive challenges, mainly in the middle or distal third leg due to limited soft tissue availability. Free tissue transfers are often the only option. Reintroduction of reliable local flaps following better anatomic understanding has opened new vistas, providing simpler yet versatile options.[12]

Muscle flaps have been traditionally favored for providing cover at exposed lower limb fracture sites with a view to providing additional blood supply and tolerance to infection.[13] The gastrocnemius and soleus have been considered the “dynamic duo” for defects of the upper and middle third leg during the past two decades,[14,15] but functional and esthetic morbidity are important disadvantages.[16] TA and FDL are essentially of limited utility for covering small defects. Being nonexpendable, the TA and FDL have been employed as segmental or split flap to cover small tibial defects.[17] Using the perforator plus concept major lengths of both muscles can be raised subperiosteally preserving most segmental perforators. With periosteal incision parallel to the tibial trunks and dissection close to perforator origins, significant sliding movement of the entire muscle *en masse* is achievable as in case 3. Extensor digitorum longus and peroneus longus with overlying skin were employed by Bloch *et al*., but they not only divided both the tendons but advocated ligation of several lower perforators making the flaps tenuous.[18] We effaced the FDL from the tibia around the fracture site without perforator sacrifice and slid it medially along with the overlying skin to provide fracture cover without function loss.

The anterolateral thigh flap has been described as an ideal soft tissue flap by Chen and Tang.[19] Our patient with carcinoma penis and metastatic lower abdominal and groin ulceration had exposed femoral vessels with risk of impending rupture. Early salvage chemo/radiotherapy was planned and required a large flap to provide stable cover. It was decided to use a perforator-based islanded skin paddle of the anterolateral thigh while retaining the tensor fascia and its vascular supply. This perforator plus flap with dual supply ensured flap survival and did not suffer loss despite early adjuvant therapy for his cancer.

Fasciocutaneous flaps are back in favor and studies prove an equal efficacy and long-term results of fracture union compared to muscle flaps. Numerous recent articles have questioned the role of musculocutaneous flaps and advocate fasciocutaneous flaps with good results.[1,12,20] There have been numerous variations in design including pedicled island flaps, both proximally and distally based, often with the need to sacrifice a major vascular trunk in a compromised limb.[21–23] These flaps have the disadvantage of shifting skin from surrounding areas, creation of a secondary defect requiring graft, and generally poor aesthetic outcomes.[24,25] Flap mobility is limited, and they may necroses at the vital area, even after flap delays. Subcutaneous neurovascular bundles are sacrificed during flap elevation, the morbidity of loss of distal limb and flap sensation and oedema considered acceptable in such situations is surely disturbing to the patient. With perforator plus flaps, it possible to preserve the neurovascular inputs to the flap and retain distal supply while avoiding secondary defects as used in Case 4. Sensate cover over the knee in Case 5 was also considered important due to the practice of kneeling during daily activities and prayers. Both patients exhibited rapid wound healing despite local sepsis with minimal flap or distal limb oedema.

Adipofascial flaps have been of limited use in lower limb trauma due to to the inherently imposed limit on their length to base ratio in order to remain viable. This restricted their utility despite favorable outcomes. The knowledge of perforator anatomy has given us the freedom to manipulate adipofascial flap designs. With perforator plus approach, adipofascial flaps are planned over Doppler identified perforator sites. Fascial dissection is done safeguarding the supplying perforators. The peninsular base of the fascia serves as an additional source of arterial input and helps in venous drainage.[3] Niranjan *et al*. in their series of fascial feeder and perforator based V-Y advancement flaps advocate preservation of cutaneous veins to prevent congestion since the fascial perforating artery is not accompanied by draining veins in all cases.[26]

The present approach of “perforator plus flap” allows fascial flaps to be used in 1:4 or larger ratios with good results. These flaps have numerous advantages over the muscle and fasciocutaneous flaps. There is no sacrifice of any muscle mass with full preservation of function in an already traumatized limb. No skin requires to be moved thus avoiding any additional defect. The flap being pliable can be turned over while perforator dissection permits a wide reach allowing coverage with esthesis.

## CONCLUSIONS

The “perforator plus” approach lends itself to diverse uses employing muscles, skin or adipofascia either alone or as composite tissues. Central to the approach is the preservation of dual vascular inputs via perforators and flap base. Minimal limitations on flap axiality, position of base, length to breadth ratios, or use in the presence of scarring/infection are the result of double blood supply. Functioning muscle and neurovascular preservation while minimizing secondary defects are advantageous outcomes, which are feasible without compromising flap requirements. This versatility allows functional preservation of muscles and nerve while permitting esthesis. Stable wound cover is achievable with minimal collateral damage.

## References

[1] Heymans O, Verhelle N, Peters S (2005). The medial adipofascial flap of the leg: Anatomical basis and clinical applications. Plast Reconstr Surg.

[2] Karacalar A, Özbek S, Özcan M (2004). Free rectus abdominis muscle flap with plantar skin graft: A combined method of aesthetic and functional reconstruction of the heel. Scand J Plast Reconstr Surg Hand Surg.

[3] Mehrotra S (2007). Perforator plus flaps. A new concept in traditional flap design. Plast Reconstr Surg.

[4] Taylor GI (2003). The angiosomes of the body and their supply to perforator flaps. Clin Plast Surg.

[5] Wei FC (2003). Perforator flap entity. Clin Plast Surg.

[6] Wong CH, Tan BK (2007). Perforator-sparing transposition flaps for lower limb defects. Anatomic study and clinical applications. Ann Plast Surg.

[7] Wong CH, Tan BK, Song C (2007). The perforator sparing buttock rotation flap for coverage of pressure sores. Plast Reconstr Surg.

[8] Desprez JD, Kiehn CL, Eckstein W (1971). Closure of large meningomyelocele defects by composite skin-muscle flaps. Plast Reconstr Surg.

[9] Moore TS, Dreyer TM, Bevin AG (1984). Closure of large spina bifida cystica defects with bilateral bipedicled musculocutaneous flaps. Plast Reconstr Surg.

[10] McCraw JB, Penix JO, Freeman BG, Vincent MP, Wirth FH (1987). Soft tissue repair of myelomeningocele defects using bilateral latissmus dorsi and trapezius musculocutaneous flaps. Ann Plast Surg.

[11] Tropet Y, Garbuio P, Obert L, Ridoux PE (1999). Emergency management of type IIIB open tibial fractures. Br J Plast Surg.

[12] Hallock GG (2000). Utility of both muscle and fascia flaps in severe lower extremity trauma. J Trauma.

[13] Gosain A, Chang N, Mathes S, Hunt TK, Vasconez L (1990). A study of the relationship between blood flow and bacterial inoculation in musculocutaneous and fasciocutaneous flaps. Plast Reconstr Surg.

[14] Hallock GG (1996). Getting the most from the soleus muscle. Ann Plast Surg.

[15] Beck JB, Stile F, Lineaweaver W (2003). Reconsidering the soleus muscle flap for coverage of wounds of the distal third of the leg. Ann Plast Surg.

[16] (1997). A report by the British Orthopaedic Association / British association of plastic surgeons working party on the management of open tibial fractures. September 1997. Br J Plast Surg.

[17] Mathes S, Nahai F (1980). Muscle flap transposition with function preservation: Technical and clinical considerations. Plast Reconstr Surg.

[18] Bloch RJ, Cordero LM, Psillakis JM, Strauch B, Vasconez LO, Hall-Findlay EJ (1998). Combined extensor digitorum longus and peroneus longus musculocutaneous flap. Grabb’s Encylopedia of flaps.

[19] Chen HC, Tang YB (2003). Anterolateral thigh flap: An ideal soft tissue cover. Clin Plast Surg.

[20] Musharafieh R, Atiyeh B, Macari G, Haider R (2001). Radial forearm fasciocutaneous free tissue transfer in ankle and foot reconstruction: Review of 17 cases. J Reconstr Microsurg.

[21] Li Z, Liu K, Lin Y, Li L (1990). Lateral sural cutaneous artery island flap in the treatment of soft tissue defects at the knee. Br J Plast Surg.

[22] Hasegawa M, Torii S, Katoh H, Esaki S (1994). The distally based superficial sural artery flap. Plast Reconstr Surg.

[23] Koshima I, Itoh S, Nanba Y, Tsutsui T, Takahashi Y (2003). Medial and lateral malleolar perforator flaps for repair of defects around the ankle. Ann Plast Surg.

[24] Ogun TC, Arazi M, Kutlu A (2001). An easy and versatile method of coverage for distal tibia soft tissue defects. J Trauma.

[25] Rajacic N, Gang RK, Krishnan J, Kojic S (2001). Lower leg reconstruction using distally based saphenous island flap. Eur J Plast Surg.

[26] Niranjan NS, Price RD, Govilkar P (2000). Fascial feeder and perforator-based V-Y advancement flaps in the reconstruction of lower limb defects. Br J Plast Surg.

